# Integrative structural analysis of *Pseudomonas* phage DEV reveals a genome ejection motor

**DOI:** 10.21203/rs.3.rs-3941185/v1

**Published:** 2024-02-21

**Authors:** Gino Cingolani, Ravi Lokareddy, Chun-Feng Hou, Francesca Forti, Stephano Iglesias, Fenglin Li, Mikhail Pavlenok, Michael Niederweis, Federica Briani

**Affiliations:** University of Alabama at Birmingham; Thomas Jefferson University; Università degli Studi di Milano; Thomas Jefferson University; Thomas Jefferson University; University of Alabama at Birmingham; University of Alabama at Birmingham; Università degli Studi di Milano

## Abstract

DEV is an obligatory lytic *Pseudomonas* phage of the N4-like genus, recently reclassified as *Schitoviridae*. The DEV genome encodes 91 ORFs, including a 3,398 amino acid virion-associated RNA polymerase. Here, we describe the complete architecture of DEV, determined using a combination of cryo-electron microscopy localized reconstruction, biochemical methods, and genetic knockouts. We built de *novo* structures of all capsid factors and tail components involved in host attachment. We demonstrate that DEV long tail fibers are essential for infection of *Pseudomonas aeruginosa* and dispensable for infecting mutants with a truncated lipopolysaccharide devoid of the O-antigen. We identified DEV ejection proteins and, unexpectedly, found that the giant DEV RNA polymerase, the hallmark of the *Schitoviridae* family, is an ejection protein. We propose that DEV ejection proteins form a genome ejection motor across the host cell envelope and that these structural principles are conserved in all *Schitoviridae*.

## INTRODUCTION

The *Escherichia coli* phage N4 and the growing list of N4-like bacteriophages represent some of biology’s most understudied bacterial viruses. Long classified as *Podoviridae* for the relatively small size of the tail apparatus (based on low-resolution negative staining electron microscopy analysis), N4-like phages are genetically and structurally profoundly different from classical *Podoviridae*. In 2020, a large-scale bioinformatics analysis revealed 115 N4-like viruses, referred to as *Schitoviridae*, after Gian Carlo Schito, the scientist who first isolated the Escherichia phage N4 in 1966 from sewers in Genoa^1^. *Schitoviridae* have grown to include eight subfamilies and numerous new genera. The number of N4-like phages characterized in recent years continues to grow, from just 33 members in 2015 to 115 viruses in 2020^1^, with many more reported in the last three years (e.g., phage AM.P2^2^, PL14^3^, VL1^4^, ΦImVa-1^5^). Phage N4 is the best-studied member of the family *Schitoviridae*. A medium-resolution asymmetric reconstruction of the N4 mature virion elucidated the basic organization of this virus^6^. Many fundamental aspects of N4-like phage biology, such as capsid assembly, tail morphogenesis, host attachment, and genome ejection/packaging, are unknown. It is also unclear if N4-phages are *cos* or *pac* packagers^7^, given that these viruses’ packaging strategy is entirely unknown. The genomic complexity of *Schitoviridae* is surprisingly vast and consistent with a genome size of ~ 75 kb, nearly twice that of most *Podoviridae* like P22 or T7, that encode about 90 ORFs, including three RNA polymerases, one of which is a massive, ~ 3,400 residues virion-associated RNA polymerase (vRNAP) unique to this family of bacterial viruses.

This study focuses on the *Pseudomonas* phage DEV, an obligatory lytic phage of the N4-like genus. DEV is part of the CKϕ4 phage cocktail that eradicates *P. aeruginosa* infections in *Galleria mellonella* (wax moth) larvae and vertebrate models^8,9^. The DEV genome encodes 91 ORFs, including a 3,398 amino acid vRNAP. The similarity of DEV to phage N4 is low, and even essential proteins like the portal or tail fibers are undetectable using conventional bioinformatic analysis. DEV receptor has not been identified yet. Most *Pseudomonas* phages studied so far exploit either the lipopolysaccharide (LPS) or the Type IV pilus (T4P) as receptors for adsorption^10^. DEV does not require T4P to infect bacteria, and LPS’s role in infection is unclear^11^. DEV cannot adsorb PAO1 mutants lacking the O-antigen polymerase Wzy, which accumulates a lipooligosaccharide decorated with a single O-antigen repeat (C + 1 LOS). On the contrary, loss-of-function *galU, algC*, or *wapH* mutants, which produce LOS species with a truncated core, are susceptible to DEV infection^10,11^.

Leveraging the power of cryogenic Electron Microscopy (cryo-EM), single particle analysis (SPA), and localized reconstruction^12^, in this study, we decipher building blocks, provide a complete annotation of all structural open reading frames (ORFs) conserved in the *Pseudomonas* phage DEV. The structural, biochemical, and functional results presented here elucidate fundamental aspects of DEV biology with general applicability to *Schitoviridae* and shed light on the existence of a genome ejection motor formed by ejection proteins after expulsion into the bacterial cell envelope.

## RESULTS

### Overview of DEV

**Figure 1:**
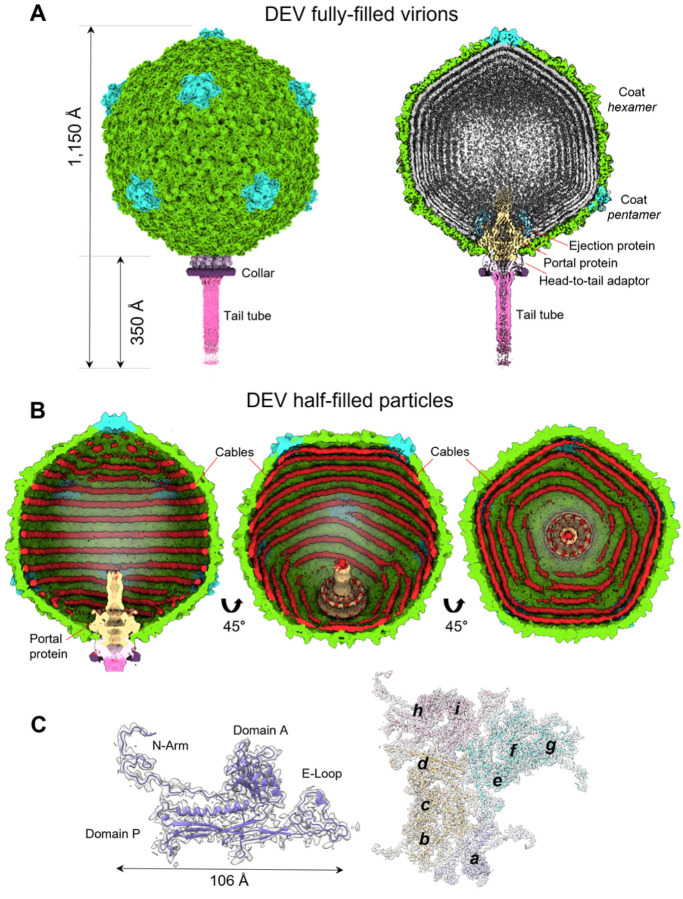
Cryo-EM analysis of the *Pseudomonas* phage DEV. **(A)** Asymmetric cryo-EM reconstruction of DEV FF virion in a side (left) and cutout (right) view. The T = 9 icosahedral shell is colored light green (hexamers) and cyan (pentons). **(B)** Asymmetric cryo-EM reconstructions of DEV HF particle. From left to right, three cutout views of the capsid are shown rotated in 45 Å increments. The cable density assigned to dsDNA is colored red. **(C)** (Left) DEV coat protein gp77 tertiary structure overlaid to a 3.3 Å C5-averaged localized reconstruction of the mature head map contoured at 5sabove background. (Right) Overview of DEV T = 9 icosahedral asymmetric unit comprising nine coat proteins labeled (*a−i*).

We purified the *Pseudomonas* phage *vB_PaeP_DEV* (Taxonomy ID: 2034344), abbreviated as DEV^8^, from PAO1 cells using cesium chloride ultracentrifugation^13^. This preparation yielded infectious virions partially contaminated with cellular debris (**Supplementary Fig. 1A**) that we used for extended data collection on a Titan Krios 300 kV transmission electron microscope equipped with a Gatan K3 detector (Table 1). 2D classification found that the specimen contained two populations of ~ 8,000 and ~ 16,000 particles, which we will refer to as half-filled (HF) and fully-filled (FF), respectively (**Supplementary Fig. 1B**). HF particles lacked bulk DNA inside the capsid, whereas FF virions were filled with DNA. Both species have a small tail apparatus (**Supplementary Fig. 1C, D**). We computed icosahedral reconstructions for either species by applying I3 symmetry and identified the unique 5-fold vertex containing the tail region using localized reconstruction and C12 averaging of the tail region. We also applied C5 symmetry at the rotation center of the unique penton to obtain a high-resolution reconstruction of the proteins forming the capsid shell. A C5–C12 aligned asymmetric virion map was then used as a reference to generate asymmetric (C1) reconstructions of FF virions (Fig. 1A) and HF particles (Fig. 1B) using a tight mask.

Phage DEV is ~ 1,200 Å in length, consisting of an ~ 800 Å icosahedral capsid with triangulation number T = 9 and a thin ~ 400 Å tail (Fig. 1A). The DEV major coat protein was built *de novo* in a 3.3 Å C5 map (Fig. 1C, **Supplementary Fig. 2A**) and identified as the gene product of ORF77 (gp77, 399 aa) (**Supplementary Fig. 3**). The coat protein, refined to a model-to-map Correlation Coefficient (CC) of 0.89 (Table 1), adopts a classic HK97 fold with an elongated N-terminal arm (res. 1–46) present in the mature virion. DEV capsid is built by 535 copies of the major coat protein (T = 9) (Fig. 1C), with one penton replaced by the portal protein. Double-stranded DNA (dsDNA) fills the interior of FF virions, forming an 8-layer core (Fig. 1A, **Supplementary Fig. 4A**). However, a medium-resolution map displayed at low contours revealed bulk density inside the capsid, looming over the portal vertex (red circle, **Supplementary Fig. 4A**). Unfortunately, this density did not align with the capsid or portal axis and could not be improved. It likely belongs to the 3,398 amino acid vRNAP, as proposed for the phage N4^6^. DEV FF virions also displayed a helical density surrounding the portal perimeter, contacting the outer two layers of dsDNA (**Supplementary Fig. 4B–D**).

### DEV half-filled particles contain DNA cables

The reconstructions of DEV obtained from HF particles and FF virions were identical from the outside, with superimposable coat protein structures. However, HF particles had peculiar rings of density laying against the capsid interior, which we termed cables, but lacked bulk dsDNA inside the capsid and the protein density surrounding the portal perimeter (Fig. 1B, **Supplementary Fig. 5A**). DEV cables most likely represent dsDNA that we estimated to account for ~ 6–6.2 kbp, equivalent to less than 10% of the DEV genome. Similar cable-like structures lining the interior of a capsid were observed in the *E. coli* phage SU10^14^. DNA cables are not readily distinguishable in DEV FF virions due to the averaging effect of DNA. However, an overlay of the HF and FF reconstructions reveals that the cables also exist in the mature virion. Purifying DEV by chloroform extraction and sucrose gradient sedimentation yielded particles devoid of bulk DNA, cables, and tails (**Supplementary Fig. 1E**) with identical coat protein lattice. This suggests the cables do not play a structural role or stabilize the capsid from the inside but perhaps are involved in capsid assembly.

DEV cables appear concentric in the C1 reconstruction (**Supplementary Fig. 5A**), which is nonetheless biased by the 5-fold symmetry used during icosahedral reconstruction. To eliminate this bias, we calculated a focused reconstruction using a mask covering one of the five angles of the unique five-fold (**Supplementary Fig. 5B**). This revealed two different cable arrangements inside the mask: in one case, two cables run along the sides of a capsid hexon with a spacing of ~ 58 Å; in the other arrangement, one cable ends before encountering a penton, generating an asymmetric Y-shaped pattern (**Supplementary Fig. 5C**). Unfortunately, due to the low number of HF particles (~ 8,000), we were not able to generate a high-resolution map and follow the complete trajectory of cables that, in the general reconstruction, remain biased by 5-fold symmetry, except in the region covered by the mask.

### Architecture of phage DEV neck and tail

**Figure 2:**
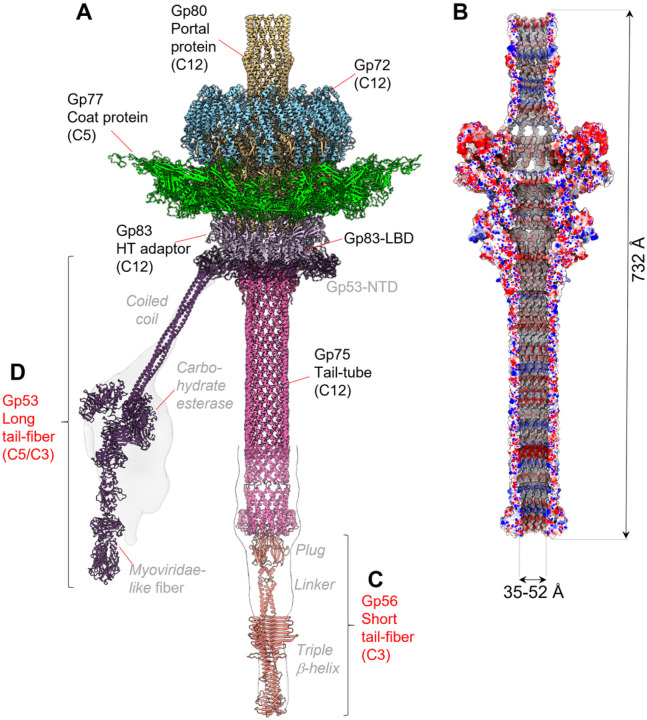
DEV tail apparatus. **(A)**. Composite ribbon diagram of DEV tail reconstructed from FF virions. Tail factors identified *de novo* in the C12 localized reconstruction include the portal protein gp80 (yellow), the ejection protein gp72 (blue), the HT-adaptor gp83 (light purple), and the tail tube gp75 (magenta). (B) Cross section of an electrostatic surface representation of the DEV tail channel. Red, blue, and white represent negative, positive, and neutral charges near the surface. **(C–D)** AlphaFold models for the short-tail fiber gp56 and long-tail fiber gp53 overlaid to low-resolution localized reconstructions shown as semitransparent surfaces. Individual tail factors are color-coded, as in panel A.

The C12 localized reconstructions of the unique vertex for FF virions and HF particles yielded 3.1 Å and 3.5 Å resolution (**Supplementary Fig. 2A**). We used these maps to build *de novo* models of the dodecameric portal protein gp80 (726 aa), twelve copies of the head-to-tail adaptor gp83 (244 aa), and twelve copies of the tail tube gp75 (321 aa) (Fig. 2A and **Supplementary Fig. 3**). In total, we built 13,212 amino acids, distributed in 36 chains (Fig. 2A), which were real-space refined to a final Correlation Coefficient (CC) of 0.89, indicating an excellent model-to-map fit (Table 1). The tail complexes reconstructed from HF and FF particles (**Supplementary Fig. 6**) are structurally identical, except that the former lacks bulk DNA inside the capsid and the helical density surrounding the portal perimeter, described below.

DEV portal protein, gp80 adopts a conserved portal fold^15^ consisting of five regions: the wing (res. 160–237 and 272–351), clip (res. 408–450), stem (res. 376–407 and 451–476), crown (res. 564–614), and C-terminal barrel (res. 620–721) (**Supplementary Fig. 3**). The inner diameter of the portal ranges between ~ 26 Å (T490) and ~ 64 Å (E566) at the bottom of the crown domain, while the narrowest diameter of the inner barrel is ~ 28 Å. A DALI^16^ search identified the related bacteriophage SPP1 portal protein (PDB:2JES) as the most similar candidate to the DEV portal protomer. The RMSD between these two proteins is 6.7 Å, and the sequence identity is only 12%. A ring of dsDNA surrounds the portal perimeter^17,18^, making direct contact with the N-arm of the portal. In the FF virion reconstruction, the portal wing interacts with dsDNA through an additional factor we identified *de novo* as gp72 (see next section) (**Supplementary Fig. 4C, D**). However, the RMSD between the portal in FF versus HF particles that lack density for gp72 is less than 0.05 Å, suggesting the latter does not affect the portal structure. Unexpectedly, DEV portal barrel^19^ was well-resolved both in FF virions (Fig. 2A) and HF particles (**Supplementary Fig. 6**), indicating the barrel is stably folded as a dodecamer in the absence of DNA. The interface between two neighboring barrel helices (res. 620–721) is stabilized by one salt bridge, 6 H-bonds, and 180 non-bonded contacts over 78 residues, significantly more bonds than in phage Sf6^20^ and P22^21^ where the portal barrel is folded only in the presence of DNA^17^ but collapses without surrounding DNA.

We also identified and *de novo* built residues 2 to 244 of DEV head-to-tail adaptor gp83 (HT-adaptor) (Fig. 2A and **Supplementary Fig. 3**). DEV gp83 consists of an N-terminal four-helix bundle extending into a C-terminal arm, as seen for P22 gp4^21,22^. It also contains a 101 amino acid lectin-binding domain (LBD) (res. 56–156) insertion in the helical bundle between α-helices H2 and H3 (**Supplementary Fig. 3**), facing outward in the tail apparatus (Fig. 2A). Instead, the gp83 C-terminal arm inserts at the binding interface between two portal protomers^22,23^. A DALI search^16^ identified the *Escherichia* phage Mu and *Klebsiella* phage KP32^24,25^ HT-adaptors as the most similar to DEV gp83, with an RMSD ~ 3 Å but only 2 ~ 7% of sequence identity.

### DEV non-contractile tail tube is 12-fold symmetric

The last tail factor we built in the C12 localized reconstruction map is the DEV tail tube (res. 2–157), the gene product of ORF75 (gp75, 321 aa), which looks like an upside-down version of the portal protein barrel (Fig. 2A, **Supplementary Fig. 3**). It attaches to the HT-adaptor and remains dodecameric without the typical 12:6 symmetry reduction seen in *Podoviridae*^20,21^. In the 3.1 Å C12 map (**Supplementary Fig. 2A**), the tail tube can only be visualized between residues 2–157, while the remaining residues 158–321 were modeled in a lower-resolution C1 map using an AlphaFold^26^ model (Fig. 2A). DEV gp75 is dodecameric with an outer diameter of 63 Å, an inner diameter of 36 Å, and approximately 330 Å in length (Fig. 2B, **Supplementary Fig. 3**). A short helix-turn-helix domain (res. 34–46) at the N-terminus of the tail tube is the only domain that contacts the HT-adaptor. The total length of the DEV tail machine from the top of the barrel to the bottom of the tail tube is ~ 732 Å, with an internal diameter between ~ 35–52 Å (Fig. 2B). A Coulombic electrostatic potential surface reveals that the DNA channel is mildly basic, especially in the barrel region and bottom part of the tail tube but contains no physical gate or constriction to prevent DNA from exiting the capsid. A distinct density for dsDNA is visible inside the tail, stronger in the top portion of the portal, tapering down toward the bottom (gray in **Supplementary Fig. 4B**). The lack of physical constriction and the continuous DNA density inside the tail tube suggests that the DEV tail is sealed by a plug at the distal tip relative to the capsid, analogous to the tail needle of phage P22^21^ and Sf6^20^.

### DEV contains two flexible fibers: the long fiber gp53 and the short fiber gp56

The high-resolution C12 localized reconstruction had minimal density for two components of the DEV tail: the fiber emanating from the tail neck, known as appendage in N4^6^, and a putative plug at the tail tube tip. Both structural components of the DEV tail can be seen in aligned micrographs and 2D-class averages (**Supplementary Fig. 7A, B**) but are smeared at high resolution. Coincidentally, two ORFs were left unassigned in the DEV genome: ORF53, whose gene product gp53 encodes a 1,090 aa protein, and ORF56, which encodes a smaller, 429 aa protein (**Supplementary Fig. 3**). We generated AlphaFold2^27^ models for both ORFs that are predicted to fold into trimeric fibers. We named gp53 the long fiber and gp56 the short fiber. To validate the existence of these fibers in DEV FF virions, we computed low-resolution focused reconstructions of the DEV tail using masks of different shapes and sizes, which revealed smeared density around the neck and at the tail tip (Fig. 2C–D, **Supplementary Fig. 7C, D**). The trimeric short fiber gp56 was docked at the tail tube tip, sealing the dodecameric tube channel (Fig. 2C, **Supplementary Fig. 7C, D**). Here, a 12:3 symmetry mismatch is plausible, as the predicted gp56 N-terminal knob fits snugly inside the tail tube. This interaction resembles the *Podoviridae* tail needle^28,29^ that seals the tail hub channel in P22^21^ and Sf6^20^, preventing DNA leakage.

### DEV long-tail fiber gp53 assembles to the tail via a 15:12 symmetry mismatch

**Figure 3:**
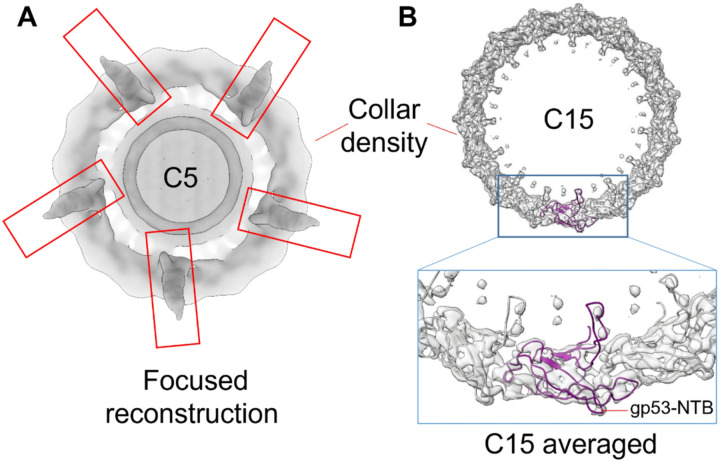
Topology and composition of DEV collar. **(A)** A low-resolution C5 map of the DEV mature virion visualized from the bottom of the tail apparatus shows density for five appendages protruding from the collar and assigned to the tail fibers gp53. **(B)** A C15 symmetrized density of the DEV collar is visualized at a high contour (5s). The AlphaFold model of the tail fiber gp53-NTB was docked into the density. Fifteen copies of gp53-NTB fill the collar density with no clashes.

Deciphering how the long-tail fiber gp53 attaches to the dodecameric tail tube was challenging. Gp53 is DEV’s equivalent to phage N4 appendages that were suggested to follow the tail C12 symmetry^6^. However, several lines of evidence suggest that DEV is not likely to contain 12 copies of gp53. First, the C12 localized map used to build the portal, HT-adaptor, and tail tube had a smeared, collar-shaped density, ~ 174 Å in diameter and ~ 27 Å thick, bonding the HT-adaptor (Fig. 1A). This density, visible at the same contour as the HT-adaptor, is featureless in our C12 map, suggesting the gp53-collar is either misaligned relative to the tail in the reconstruction or averaged out by applying incorrect rotational symmetry. Second, a localized reconstruction of DEV putative appendages revealed ve elongated densities emanating outward from the tail (Fig. 3A, **Supplementary Fig. 7C**). Third, AlphaFold2^27^ predicts gp53 to fold into a trimeric fiber containing an N-terminal β-barrel (NTB, res. 1–91), flexibly connected to a trimeric coiled-coil domain (res. 115–250), a carbohydrate esterase-like domain (res. 265–656) and a *Myoviridae*-like fiber (res. 694–1090) (**Supplementary Fig. 7C**). We then hypothesized that the DEV gp53-collar has higher symmetry than the dodecameric tail. To test this idea, we expanded C12-aligned particles along the Z-axis to C24 and C30, followed by an asymmetric search without sampling with a donut-like tight mask. The 3D classification was not well resolved for C12 expanded particles but sufficient to identify 15 repeated density blobs. One class from the C30 symmetry expansion consisting of 8,000 particles after removing duplicates could be refined, yielding maps of the C15 symmetrized and asymmetric collar to 4.7 Å (Fig. 3B) and 6 Å resolution (**Supplementary Fig. 8A**), respectively.

To model the long-tail fiber, we docked 15 copies of gp53 NTB (**Supplementary Fig. 2, Supplementary Fig. 3**) into the C15 and C1 collar densities and real space refined to a final CC = 0.75 and 0.82, respectively. The fitting is convincing, with 15 β-barrels (res. 1–91) making head-to-tail contacts and projecting a 30 amino acids loop (res. 92–114) toward the HT-adaptor (Fig. 3B). The prominent gp53 trimeric coiled-coil (res. 115–250) fits remarkably well with the elongated density projecting outward from the DEV tail (Fig. 2D), positioning the flexible esterase-like domain and *Myoviridae*-like fiber in the smeared density projecting away from the tail (Fig. 2D, **Supplementary Fig. 7C**). This model suggests that 15 gp53-NTBs form a collar around the tail, generating a 15:12 symmetry mismatch with the HT-adaptor C12 ring (**Supplementary Fig. 8B**), like in the podophage GP4^30^. An unstructured linker in gp53 spanning residues 92–114 (**Supplementary Fig. 3**) provides the flexibility to connect 15 gp53 NTBs with five trimeric coil-coiled domains, positioning the esterase-like domains and fiber regions outward from the tail tube. These domains are likely involved in LPS interaction and phage adsorption.

### The long fiber gp53 is necessary but not sufficient to infect P. aeruginosa

**Figure 4:**
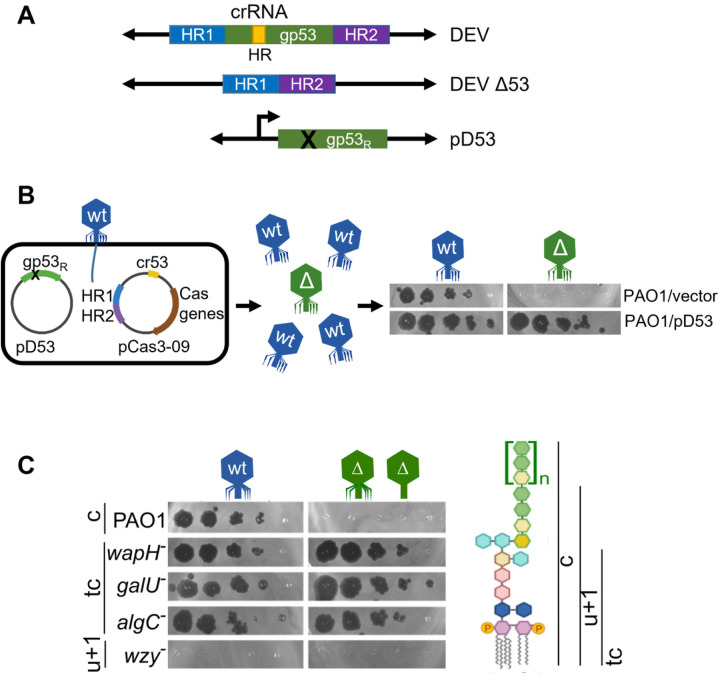
Role of DEV long fiber gp53 in host attachment. **(A)** Structure of the gp53 locus in DEV and DEV Δ53 phages and the pD53 plasmid. crRNA HR, region targeted by cr-RNA53 expressed by pCas3-09; HR1 and HR2, homology regions cloned in pCas3-09 plasmid; gp53_R_, cr-RNA53 resistant gp53 allele cloned in pD53. **(B)** Outline of DEV mutagenesis. Infection with DEV of PAO1 carrying pCas3-09 and pD53 produces a genetically mixed phage progeny with wt (in blue) and Δ53 (in green) virions. Unlike *gp53*^+^ DEV, Δgp53 mutants grow on PAO1 carrying pD53 (pD53) but not on PAO1 containing the empty vector pGM931 (EV). **(C)** DEV Δgp53 growth on mutants with LPS defects. Serial dilutions (x 10) of DEV or Δgp53 were replicated on PAO1 and the indicated PAO1 mutants with defective LPS. On the right, the structure of PAO1 LPS (c, capped) and the LPS portions present in the mutant LPS variants. tc, truncated core; u+1, uncapped LPS + one O-antigen repeat.

To characterize the function of DEV gp53 long-tail fiber and discern its role in phage adsorption, we generated a DEV gp53 deletion mutant (DEV Δ53; Fig. 4A). To obtain the mutant phage, we exploited a minimal Cascade-Cas3 system (Type I-C), generating large deletions whose boundaries can be specified by a homology-directed repair (HDR) template^31^. DEV genome editing was performed in *P. aeruginosa* PAO1 strain containing the plasmids pCas3-09 and pD53 (editing strain; Fig. 4B and Table 2). pCas3-09 is a pCas3cRh derivative expressing all components of the Cascade-Cas3 (Type I-C) system (i.e., *cas3, cas5, cas8*, and *cas7*;^31^) and a gp53-specific gRNA (cr-RNA53). pCas3-09 also carried the HDR template, i.e., two fused DNA fragments corresponding to the gp53 flanking regions in the DEV genome. As expected, DEV did not grow on PAO1 expressing the cr-RNA53 Cascade complex (**Supplementary Fig. 9A**). pD53 contained the gp53 gene with silent mutations in the region recognized by the cr-RNA53 (gp53R allele; Fig. 4A and B) that made pD53 resistant to cr-RNA53-targeted Cas3 digestion. Expression of gp53R from pD53 complemented the Δ53 mutation so that even phage genomes lacking the gp53 gene were packaged into complete virions. The editing strain was infected with DEV, and the lysate was plated on the permissive PAO1/pD53 strain. Almost all phages that were unable to reproduce in the absence of pD53 (Fig. 4B) contained the expected gp53 deletion in their genomes (**Supplementary Fig. 9B**). The DEV Δ53 mutant did not reproduce in PAO1 but, surprisingly, it grew in *algC, galU*, and *wapH* mutant strains, which all make uncapped LOS species lacking the O-antigen^11,32^ (Fig. 4C). Thus, gp53 is essential for PAO1 infection in the presence of smooth-type, O-antigen capped LPS but dispensable if rough-type, uncapped LOS is produced. Both DEV and DEV Δ53 mutant did not grow in the *wzy* mutant (Fig. 4C), which accumulates a LOS form capped with a single O-antigen repeat^11,32^, suggesting that in the *wzy* mutant, no receptor is present/accessible to the phage.

### An ejection protein surrounds the DEV portal protein

**Figure 5:**
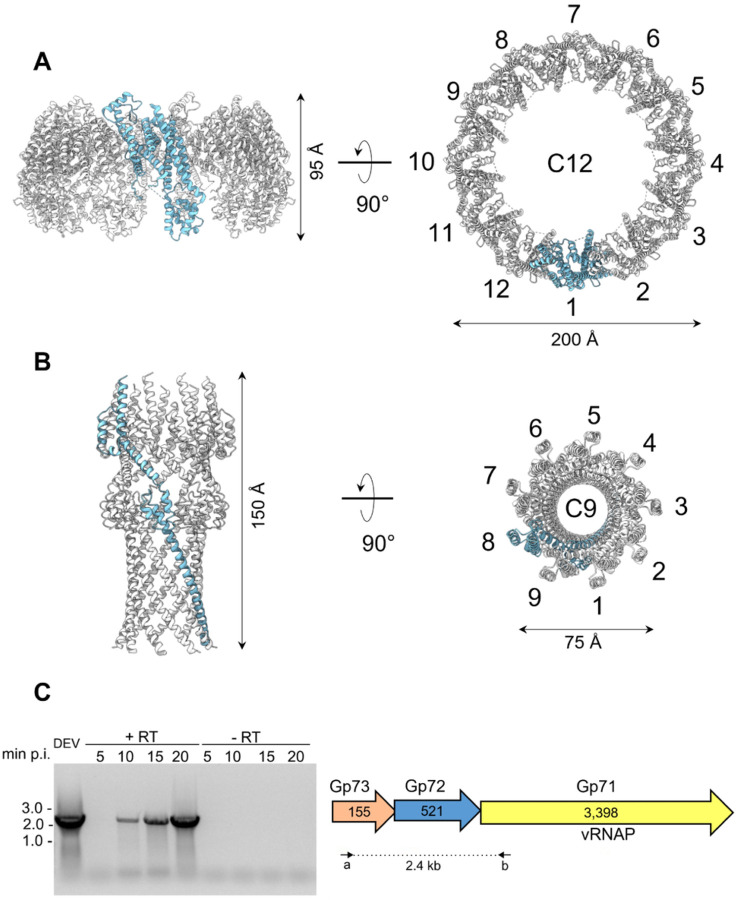
Quaternary structures of DEV ejection protein gp72 pre- and post-ejection. **(A)** The quaternary structure of DEV gp72 from FF virions determined in situ. Twelve gp72 subunits surround the portal protein, generating a ~200 Å wide ring. **(B)** Cryo-EM structure of the recombinant nonameric gp72 determined at 3.65 Å resolution in the post-ejection conformation. In panels **A** and **B**, only one protomer is colored in cyan, whereas all other subunits are light gray. **(C)** DEV gp71, gp72, and gp73 genes are co-transcribed as an operon. (Left panel) Agarose gel electrophoresis of RT-PCR products. RNA samples extracted from PAO1 cultures at different time points post-infection (p.i.) with DEV (e.g., 5, 10, 15, 20 minutes) were reverse-transcribed (+RT) or not (negative control, −RT) and used as templates for PCR amplification. Migration of MW (kb) markers is shown on the left. (Right panel) Schematic diagram of DEV ORFs encoding gp71, gp72, and gp73. Arrows represent the position of oligonucleotides used for amplification, yielding a 2.4 kb long amplification product.

The initial localized reconstruction of the FF virion revealed tubular density surrounding the portal wings (**Supplementary Fig. 4C**). To improve this density, we computed a focused reconstruction using a tight mask, which only covers the helical region, and applied C12 rotational symmetry, yielding a 4.0 Å map (**Supplementary Fig. 2A, Supplementary Fig. 4D**). We built this density *de novo* and identified the protein surrounding the DEV portal as the gene product of ORF72 (gp72, 521 aa). The modeled structure (res. 116–495) lacks residues 1–115 and 496–521, which are likely too flexible to be aligned. Applying 12-fold symmetry, we generated a dodecameric model of gp72 (Fig. 5A), consisting of 4,740 residues, which we real-space refined to a final CC = 0.7 (Table 1). Twelve copies of gp72 form a 200 Å-wide dodecameric ring, concentric to the portal protein. Bioinformatic analysis revealed that DEV gp72 has 7% identity and 15% similarity to phage T7 ejection protein gp15 that forms a periplasmic tunnel (PT) in the E. coli cell envelope after ejection^33,34^.

The hallmark of ejection proteins is that they exist in a pre-ejection conformation in the virion, before genome ejection, and a post-ejection conformation inside the host cell envelope: we referred to these proteins as conformational gymnasts in a recent review^35^. Previous work on T7 ejection protein gp15 revealed that the recombinant protein expressed in bacteria^36^ adopts a post-ejection conformation, distinct from the metastable conformation seen in the mature virion before ejection (pre-ejection conformation)^37^. To test the hypothesis that DEV gp72 is an ejection protein, we cloned, purified, and solved a cryo-EM structure of the recombinant gp72 protein (**Supplementary Fig. 10A, B**). We found that gp72 assembles into a nonameric elongated channel, 80 Å in width, containing a 25 Å wide lumen large enough to fit dsDNA (Fig. 5B), morphologically similar to gp15 PT. Thus, the quaternary structure difference between gp72 in virion and recombinant involves a tertiary structure folding and a change in stoichiometry from 12 to 9 subunits.

In phage T7 and many *Podoviridae* studied to date^35^, the ejection protein gp15 is neighboured by a smaller gene encoding the membrane protein gp14 and a much larger gene encoding gp16 (> 1,300 residues), implicated in genome ejection^38^. Together, the genes encoding gp14, gp15 and gp16 form an operon. We then asked if the two ORFs in DEV surrounding gp72, known as gp71 and gp73, also encode ejection proteins and whether gp72 is part of an operon^35^. RT-PCR of DEV-infected cells revealed that gp72 and the flanking gp71 and gp73 are expressed as a polycistronic mRNA ~ 10 minutes after DEV infection of PAO1 (Fig. 5C). Remarkably, DEV gp71 happens to be the giant vRNAP that in the related phage N4 is known to be injected in *E. coli* during infection^39^.

### Structure of DEV ejection protein gp72:gp73 complex

**Figure 6:**
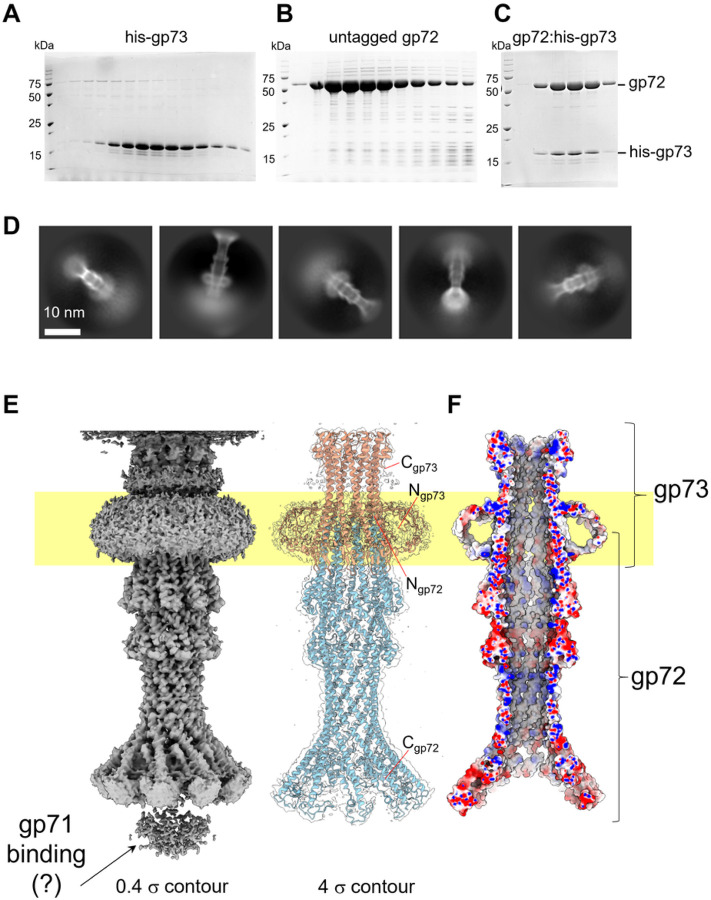
DEV ejection proteins gp72 and gp73 form a tube-shaped complex. SDS-PAGE analysis of purified **(A)** hisgp73 solubilized from membranes; **(B)** gp72 expressed under native conditions; **(C)** gel filtration fractions containing the gp72:gp73 complex. **(D)** Representative 2D class averages of the gp72:gp73 complex. **(E)** 3D reconstruction of the gp72:gp73 complex visualized at low (left) and high (right) contours. The atomic models of gp73 and gp72 are overlaid to the semitransparent density calculated at 3.15 Å resolution. In yellow is the putative position of the bacterium’s outer membrane. **(F)** The cross-section of an electrostatic surface representation of gp72:gp73 shows the lumen and surface charge inside the channel. Red, blue, and white represent negative, positive, and neutral charges near the surface.

Phage ejection proteins form a channel in the bacterial cell envelope spanning the outer membrane (OM), periplasm, and inner membrane (IM) projecting into the host cytoplasm^35^. We have established that DEV gp72 resembles a PT, so we reasoned that gp73 may form an OM pore (OMP) similar to T7 gp14 that has pore-forming activity in lipid bilayers^40^ or P22 T7 that partitions into lipid nanodiscs *in vitro*^21^. This hypothesis was bolstered by transmembrane prediction servers, MemBrain^41^ and DAS^42^, which predict a transmembrane α-helix spanning residues 77–91 in gp73 (**Supplementary Fig. 11A–C**). *In vitro*, recombinant gp73 (M.W. 17.7 kDa) was completely insoluble, which prompted us to purify the protein from the membrane fraction of DEV-infected *P. aeruginosa* PAO1 using n-dodecyl β-D-maltoside (DDM) (Fig. 6A). To test if detergent-solubilized gp73 was active, we mixed it with gp72 (Fig. 6B), which is water-soluble. Size exclusion chromatography (SEC) indicated that the two recombinant proteins form a complex (Fig. 6C) that we successfully vitrified. An initial dataset of gp72:gp73 collected on a 200 kV cryo-electron microscope gave convincing evidence that the two proteins form a tube-like structure, more extended than gp72 alone. We next collected a large dataset on a 300 kV Krios electron microscope and determined a 3D reconstruction of the gp72:gp73 complex by single particle analysis (Fig. 6D, E) at a resolution between 3.15–6 Å (Table 1, **Supplementary Fig. 2C**). The structure revealed a striking 9-fold rotational symmetry that allowed us to dramatically improve the experimental density by applying C9 symmetry. Overall, we built *de novo* residues 1–155 of gp73 and residues 25–331 of gp72, which were real-space refined to a final Correlation Coefficient (CC) of 0.89, indicating an excellent model-to-map fit (Table 1).

**Figure 7:**
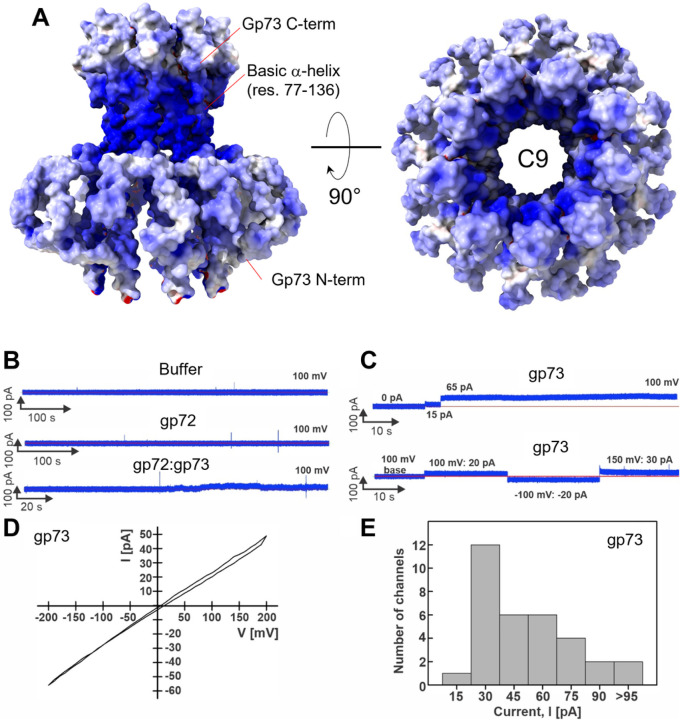
Lipid bilayer experiments with purified DEV ejection protein gp73. **(A)** The electrostatic surface representation of nonameric gp73 reveals a significant positive charge, mainly in the a-helical core. **(B–C)** Lipid bilayer experiments were performed at 100 mV applied potential in diphytanoylphosphotidylcholine (DPhPC) membranes bathed in 1 M KCl, 10 mM HEPES, pH 7.4 electrolyte. The protein samples were added to the grounded trans side of the cuvette, which had 100 μm SU-8 aperture. **(B)** Representative current traces. Top: 15 μl protein buffer in the cuvette. Six membranes were recorded with 1 – 15 μl of the protein buffer, and no activity of the buffer was observed. Middle: gp72 current trace. Seven membranes with up to 24 μg of gp72 in the cuvette were recorded, and no channel activity was observed. Bottom: gp72:gp73 complex. 19 membranes were recorded, and only one shown here had 10 – 20 pA fluctuations around the baseline when 10 μg of protein sample was in the cuvette. **(C)** Representative current traces of gp73. Top: Two insertions of gp73 (750 ng protein in the cuvette) with amplitudes of 15 pA, and 65 pA. Bottom: Continuous current trace of a single gp73 insertion (900 ng in the cuvette) at indicated voltages. **(D)** Current-voltage curve of one gp73 pore inserted in the DPhPC membrane at a voltage range of −200 to 200 mV. **(E)** Histogram of single-channel current amplitudes of gp73 at 100 mV. A total of 33 channels were observed with a mode current of 30 pA.

The membrane protein gp73 forms a cap onto which gp72 inserts (Fig. 6E), generating a trumpet-shaped complex ~ 300 Å in length with an internal diameter of 35 Å (Fig. 6F). Gp73 and gp72 mainchains run antiparallel, with the N-termini of the two proteins located in relative proximity and the C-termini displaced by more than 200 Å (Fig. 6E). Extensive intermolecular interactions between gp73 residues 64–93 and gp72 N-terminal helix (res. 25–54) stabilize the gp72:gp73 nonameric interface (~ 67,944 Å^2^), comprising 24 hydrogen bonds and 723 non-bonded interactions (**Table S2**). Strikingly, the gp73 long a-helix spanning residues 77–137 makes up the inner channel and the region facing the bacterial outer membrane, which is very positively charged (Fig. 7A). We speculate that a positive charge may favor insertion in the negatively charged lipid A building the OM^43^. The soft density around the gp73 N-termini (Fig. 6E) is likely disordered DDM used for solubilization, suggesting this may be part of the gp73 is also hydrophobic and may interact with lipids, perhaps by inserting laterally in the OM instead of penetrating through like the central a-helix. On the opposite tip, gp72 flowers like a trumpet, possibly to provide a binding domain for gp71. Notably, the gp73-bound structure of gp72 has an additional 90 residues visible at the C-terminus relative to the isolated gp72 (e.g., the last residues in density are E331 and D224, respectively) (Fig. 6E, **Supplementary Fig. 10C**). This suggests that gp73 binding stabilizes the overall quaternary structure of gp72, and, similarly, gp71 may also stabilize gp72 C-terminal residues 332–521, invisible in our reconstruction. The structure of gp71 remains unknown. An AlphaFold^26^ model suggests a fully structured ~ 320 kDa polypeptide chain consisting of three domains, of which the RNA pol encompasses residues 820–1,900. The other predicted domains, the N-terminal domain (res. 1–760) and C-terminal domain (res. 1,900–3,398), are highly structured and enriched in α-helices but lack similarity to known structures.

### Lipid bilayer experiments reveal that DEV gp73 is a membrane-spanning pore protein

A phage ejectosome requires a continuous channel from the phage head to the bacterium cytoplasm. The cryo-EM of the gp73 revealed a hollow nonameric channel with an internal diameter wide enough to accommodate DNA (~ 25 Å) and hydrophobic helices flowering outbound to gp72 and extensive basic residues, possibly involved in binding lipid A phosphate groups in the bacterial outer membrane (Fig. 7A). To examine the channel-forming properties of DEV ejection proteins gp72 and gp73, we performed lipid bilayer experiments as described previously^36,40,44^. To this end, the gp73 protein was solubilized in a buffer containing 0.2 mM DDM, while the gp72 and gp72:gp73 complex were solubilized in a buffer without detergent. As a negative control, the DDM buffer alone did not change the baseline current in lipid bilayer experiments (Fig. 7B, **top panel; Supplementary Table S3**), demonstrating that the detergent alone does not perturb the lipid membrane. Like the buffer, purified gp72 (Fig. 7B, **middle panel**) and gp72:gp73 complex (Fig. 7B, **bottom panel**) did not show channel activity in our experiments. We observed one membrane out of 19 with current fluctuations of 10 to 20 pA when 10 μg of gp72:gp73 complex was added (Fig. 7B, **bottom panel; Supplementary Table S3**). These results are inconsistent with a stably populated pore in the lipid bilayer, suggesting that the gp72:gp73 complex does not form a stable pore in the membrane. In contrast, the addition of purified gp73 protein in amounts ranging from 0.3 μg to 2.6 μg resulted in a stepwise current increase indicative of insertions of open, water-filled channels into the lipid bilayer (Fig. 7C, D **and Supplementary Fig. 12A, B**). We analyzed 33 gp73 channels in 17 different membranes using different protein fractions after gel filtration and observed a wide distribution of open pore current at 100 mV (Fig. 7E, **Supplementary Table S3; Supplementary Fig. 12C–F**). The mode value of open pore current was 30 pA, while some channels had a greater current amplitude ranging from 45 to 95 pA (Fig. 7E). These experiments conclusively demonstrate that the gp72-gp73 complex forms a membrane-spanning structure capable of translocating DNA across the outer membrane of *P. aeruginosa*.

## DISCUSSION

Infections caused by the Gram-negative pathogen *P. aeruginosa* are a leading cause of morbidity and mortality worldwide. Phage therapy against *P. aeruginosa* has gained attention as a promising therapeutic weapon, especially in the fight against cystic fibrosis-related infections^45,46^. The N4-like phage DEV is part of an experimental phage cocktail to eradicate *P. aeruginosa* infections *in vivo*^8,9^. In this study, we determined a complete structural atlas of all phage DEV structural factors and elucidated fundamental aspects of DEV biology. We resolve a new symmetry mismatch in the tail apparatus, identify two putative fibers, and decipher three ejection proteins. As *Schitoviridae* genomes are largely unannotated and many ORFs have unknown functions, our work paves the ground for the facile identification of structural components when a new *Schitoviridae* phage is discovered.

The mechanisms of *Schitoviridae* attachment to bacteria are poorly understood. N4 binds the *E. coli* membrane protein NfrA^47^ via its tail sheath^48^, which surrounds the tail tube. However, this attachment mechanism is unlikely for DEV that lacks a tail sheath. Based on all data presented here, we hypothesize that gp53 and gp56 mediate DEV adsorption to the *P. aeruginosa* surface. We posit that the long fiber gp53 binds the highly abundant O-antigen that serves as a primary receptor. The short fiber gp56 contacts a secondary receptor in the OM that triggers its detachment from the tail tube, prompting genome ejection.

In previous work, we postulated that DEV adsorption to *P. aeruginosa* PAO1 and other strains with LPS of the same serotype might involve two receptors: the O-antigen and another unidentified receptor^49^. The lack of infection of the DEV Δ53 is consistent with the hypothesis that gp53 is the receptor-binding protein involved in O-antigen recognition. The DEV phage may exploit gp53-dependent O-antigen binding to approach the second receptor when infecting PAO1. Conversely, gp53 is dispensable for adsorption to PAO1 mutants with a truncated LPS core, suggesting that long-tail fibers are not needed if the LPS does not mask the second receptor. Thus, like T5 and other phages, DEV can bind directly to the second receptor when accessible, using another receptor-binding protein, most likely the short-tail fiber, gp56, that binds receptors in other podoviruses^50–52^. It remains unclear why the *wzy* mutant, which produces a LOS form capped with a single O-antigen repeat^11,32^, is resistant to both DEV and Δ53. We speculate that the *wzy* LOS may be too short to allow gp53-dependent DEV binding but long enough to mask the second receptor.

*Schitoviridae* have large DNA genomes, but how these viruses eject their DNA into bacteria is unknown. In small *Podoviridae* like T7 and P22, ejection proteins form a DNA-ejectosome^33,36^ that extends the short tail, allowing DNA passage through the cell envelope^34,35,53^. However, ejection proteins have not been mapped in N4 or other members of the *Schitoviridae* family^1^. In this work, we found that the DEV portal is surrounded by 12 copies of a helical protein that we built *de novo* and identified as gp72. The position in the virion and helical fold of DEV gp72 is similar to the recently characterized gp45 cargo protein C1 found in the CrAssphage ΦcrAss001^54^. We demonstrate that DEV gp72 forms a 200 Å-wide dodecameric ring, concentric to the portal protein. Cryo-EM reconstructions show that the recombinant DEV gp72 and gp73 proteins assemble into a long channel large enough to accommodate dsDNA. Lipid bilayer experiments demonstrate that gp73 can insert into membranes and form stable pores. Together, these results provide convincing evidence that gp73 and gp72 form a continuous channel that crosses the outer membrane and the periplasmic space of *P. aeruginosa*. Our data suggest that gp73, gp72, and gp71 are DEV ejection proteins functionally equivalent to *Podoviridae* gp14, gp15, and gp16. Importantly, gp71 is DEV’s equivalent to N4 vRNAP, which is injected in E. coli during infection^39^. This enzyme is responsible for an early burst of RNA synthesis, observed immediately after N4 infection, even after inhibiting the host RNA pol^55^. Notably, N4 vRNAP initiates transcription of early gene products gp1 and gp2 that act as cofactors for the heterodimeric polymerase N4 RNAPII (gp15:gp16) required for transcription of middle genes^39^. Among these transcripts, N4SSB redirects the host RNA pol to late promoters, mediating the expression of late genes involved in virion assembly, DNA replication, packaging, and host lysis. Interestingly, N4 vRNAP transcribes efficiently only denatured N4 DNA *in vitro* but is entirely inactive on native N4 DNA, pointing to an involvement of host DNA gyrase to introduce negative supercoils into the phage genome and drive transcription of early promoters. Thus, we propose that the DEV entire genome is actively pulled into the host by the combined action of three sub-motors: first, the ejected vRNAP, gp71, required for early gene transcription, possibly aided by host gyrases that introduce negative supercoils into the phage genome; second, the transcription activity by DEV second RNA polymerase, RNAPII; lastly, the host RNA polymerase.

The results presented in this paper allow us to formulate a model for how DEV attachment to the *Pseudomonas* surface triggers the ejection of the phage genome through the tail tube^56^. We envision three steps in this infection process.

**Figure 8:**
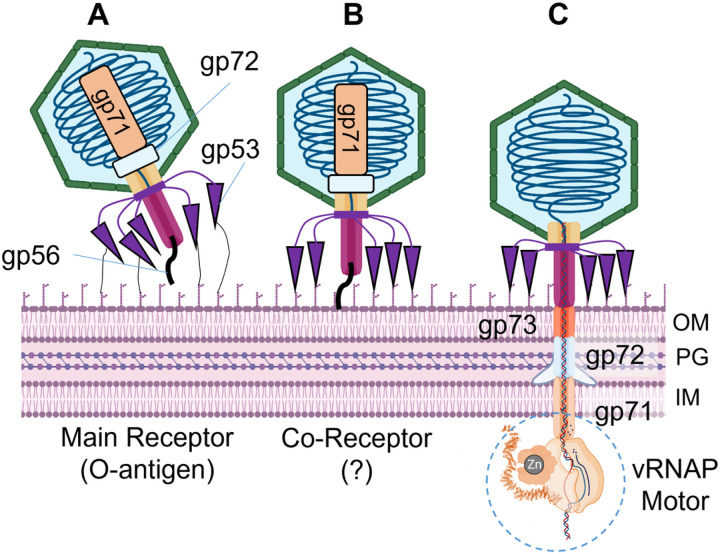
Model for DEV absorption onto *P. aeruginosa* surface and genome ejection. Three proposed steps of infection are shown: each step is accompanied by distinct conformations of the long and short-tail fibers. **(A)** DEV interacts with the host O-antigen through flexible long-tail fibers (gp53), possibly reorienting the virion to land perpendicular to the OM. **(B)** The short-tail fiber gp56 interacts with a secondary receptor in the bacterium OM, triggering a conformational change that releases the short fiber. **(C)** The ejection proteins gp73, gp72, and gp71 are expelled into the bacterium cell envelope where gp72 forms an OM pore, gp73 spans the periplasm, and gp71 crosses the IM, projecting a large vRNAP motor into the bacterial cytoplasm, that begins pulling the viral genome inside the host. PG = peptidoglycan

**Step 1** (Fig. 8A). DEV long-tail fibers are flexible, stochastically fluctuating to enhance the chance of encountering LPS. The association of gp53 with the host O-antigen, possibly mediated by the carbohydrate esterase-like domain (res. 265–656) and *Myoviridae*-like fiber (res. 694–1090), tethers the phage closer to the host surface, likely inducing the sequential attachment of all five long-tail fibers. We did not detect LPS hydrolase activity in isolated DEV virions, suggesting the long-tail fibers only function by adsorbing the virion to the cell surface instead of shaving off LPS as in P22^57^. The association of multiple long-tail fibers with the LPS may reorient the virion to land perpendicular to the OM, as observed for phage P22^53^.

**Step 2** (Fig. 8B). After the virion has attached to the host surface, the short-tail fiber, which also functions as a tail plug, encounters a secondary receptor, likely an outer membrane protein shielded by LPS and thus inaccessible from the outside. Similar to phage DEV, recent data suggests that N4 also exploits two receptors to infect its *E. coli* host: the outer membrane protein NfrA^48^ and an *N*-acet2ylmannosamine-based surface carbohydrate produced and exported to the cell surface of *E. coli* in a c-di-GMP-dependent manner^58,59^. We hypothesize that the interaction of gp56 with the secondary receptor transmits a mechanical signal that releases the short-tail fiber. Analogous to the tail needle gp26 in P22-like phages^29^, the short-tail fiber could be ejected into the host or removed upon association with the receptor.

**Step 3** (Fig. 8C). Following the release of gp56, the ejection proteins gp73, gp72, and gp71 are expelled into the bacterial cell envelope where gp72 forms an outer membrane pore, analogous to P22 gp7 and T7 gp14. Gp73 spans the periplasm, like the periplasmic tunnel gp15 in T7, and gp71 crosses the IM, projecting a large RNA pol motor into the bacterial cytoplasm. As observed for phage T7, we determined a reduction of the gp72 oligomeric state from dodecameric inside the virion to nonameric after ejection^40^. In T7, the periplasmic tunnel gp15 is octameric in capsid and hexameric after ejection. Thus, a loss of ejection protein subunits accounts for the challenging refolding-coupled assembly of the DNA-ejectosome in *Schitoviridae*, as suggested for small *Podoviridae*^60^. However, the major difference is that *Schitoviridae* have a much larger genome (~ 75 kbs) and carry a vRNAP that must become transcriptionally active to promote successful infection^39^. This function is exerted by the host RNA pol in small *Podoviridae* that rely chiefly on the host transcription machinery to eject their smaller genomes.

In summary, we have deciphered the architecture and design principles of a prototypical N4-like phage used in an experimental phage therapy cocktail. We propose that the structural principles elucidated in this work are conserved in other *Schitoviridae* of the widespread N4-like family^1^. It is also possible that the anatomy of DEV ejection proteins, the interplay with virion-associated and encoded RNAPs, and their role in genome delivery will be conserved in crAss-like phages, the most abundant viruses in the human gut^54^. We anticipate that the 3D-atlas of DEV structural proteins described in this paper will allow the mapping of resistance mutations and facilitate the identification of ORFs in related *Pseudomonas* phages used in phage therapy cocktails.

## METHODS

### Bacteria, bacteriophages and plasmids

*P. aeruginosa* strains, bacteriophages, and plasmids are listed in Table 2; oligonucleotides are in **Table S1**. *P. aeruginosa* genome coordinates refer to PAO1 strain, NCBI RefSeq NC_002516.2. Plasmids were constructed in *Escherichia coli*, and their relevant portions were sequenced before they were transferred into P. aeruginosa by transformation. pCas3cRh^31^ is a pHERD30T derivative and was purchased from Addgene. pCas3-01 was obtained by cloning the annealed 3697 and 3698 primers in the pCas3cRh BsaI restriction site. pCas3-09 and pD53 were constructed by assembling DNA fragments using the NEBuilder HiFi DNA Assembly Master Mix (New England Biolabs). pCas3-09 derives from the assembly of pCas3-01 linearized with BstZ17I with two fragments obtained by PCR on DEV DNA with primers 3807–3840 and 3808–3841. pGM2148 carries the DEV gp53 gene under the control of *araBp* promoter and was obtained by assembling pGM931^61^ digested with *KpnI* and the gp53 gene amplified by PCR on DEV DNA with primers 3804–3805. pD53, which carries crRNA53-resistant gp53, was obtained by PCR amplification of pGM2148 with primers 3859–3955 and ligation of the amplicon with the KLD Enzyme Mix (New England Biolabs). The genes encoding gp72 and gp73 were cloned by PCR from DEV DNA between restriction sites NdeI and XhoI in pET30b and pET22b, respectively. Gp73 was cloned with C-terminal 6x His tag, while gp72 was untagged. Bacterial cultures were grown in Lysogeny Broth (LB) at 37°C. Cultures of bacterial strains carrying plasmids were supplemented with 100 μg/ml ampicillin, 50 μg/ml gentamicin, 300 μg/ml carbenicillin, 0.2% arabinose (w/v), and 0.1% rhamnose (w/v) when needed.

### Purification of DEV virions for cryo-EM

DEV was prepared for cryo-EM analysis essentially as described^13^ with minor modifications (standard protocol) or upon chloroform extraction (modified protocol). In brief, *P. aeruginosa* strain PAO1 was grown at 37°C to OD_600_ = 0.5, corresponding to about 2.5 × 10^7^ cfu/ml in LB and infected with DEV phage^8^ (GenBank MF490238.1) at a multiplicity of infection (m.o.i.) of 0.001. Growth was continued until cell lysis was detected as a drop in the OD_600_. The lysate was incubated 30 min at 37°C with DNase I and RNase A (1 μg/ml each) and centrifuged 20 min at 5,000 × g. After supernatant filtration through a 0.45 μm filter, 58 g l^− 1^ NaCl and 105 g l^− 1^ polyethylene glycol (PEG) MW 6K were dissolved in the supernatant. The solution was incubated 16 hours at 4°C, and the phage particles recovered by centrifugation at 4°C and 20,000 × g for 30 min. The pellet was resuspended in TN buffer (10 mM Tris-HCl, 150 mM NaCl pH 8), and phages were purified according to either standard or modified protocol. In standard protocol, the mixture was stratified on the top of a cesium chloride step gradient from 1.3 to 1.6 gm/cc cesium chloride, top to bottom, formed in polyallomer ultracentrifuge tubes for Beckman rotor SW41, and centrifuged at 100,000 × g for 120 minutes at 4°C in a Beckmann Optima XE-90 ultracentrifuge using a SW41 rotor. The phage bands, which usually sediment at the 1.4 gm/cc step, were transferred into polyallomer tubes for SW41 Beckman rotor. The tubes were filled with a solution of cesium chloride 1.4 gm/cc in TN buffer and centrifuged 16 hours at 150,000 × g. After centrifugation, the phage bands were collected, dialyzed 2 × for 20 minutes against water and 16 hours against TN buffer, filtered through 0.22 μm filters, and stored at 4°C. In the modified protocol, the mixture was mixed with 0.5 volumes of chloroform and centrifuged for 15 min at 4,500 × g. The upper phase was recovered, centrifuged on the 1.3 to 1.6 gm/cc cesium chloride step gradient, and dialyzed against water and TN buffer as described before. The phage mixture was layered onto a 10–40% (w/v) sucrose gradient in TN buffer and centrifuged 1 h at 210,000 × g at 4°C using a SW41 rotor. After centrifugation, 0.4 ml fractions were collected, and their OD_260_, OD_280_ and OD_320_ were measured and plotted. The fractions corresponding to absorbance peaks were dialyzed, filtered through 0.22 μm filters, and stored at 4°C.

### DEV mutagenesis

A culture of *P. aeruginosa* PAO1 carrying plasmids pCas3-09 and pD53 was grown in LB supplemented with gentamicin, carbenicillin, arabinose, and rhamnose to OD_600_ = 0.1. The two plasmids are maintained in the same cell, although they share the same replicon, in the presence of gentamycin and carbenicillin. 1 ml was infected with DEV phage at a m.o.i. of 10 and incubated at 37° for 5 min static and 40 min with agitation. The mixture was serially diluted and plated to obtain single plaques using PAO1/pD53. After overnight incubation at 30°C, some plaques were analyzed by replica plating as described^49^ on PAO1/pD53 (permissive strain) and PAO1/pGM931 (non-permissive strain). In replicate experiments, we found that between 7 and 24% of plaques were formed by phages unable to reproduce in the absence of pD53. Decreasing the m.o.i. to 1 did not improve editing efficiency. The plaques formed by pD53-dependent phages were controlled by PCR with oligonucleotides 3806 and 3809 to confirm the presence of the gp53 deletion.

### RT-PCR analysis of DEV RNA

*P. aeruginosa* PAO1 cultures were grown in LB at 37°C up to OD_600_ = 0.8 and infected with DEV at a m.o.i. of 5–6. 3 ml samples were collected at 0-, 10-, 15- and 20-minutes post-infection for RNA extraction. RNA extraction was performed by phenol-chloroform treatment of cell lysates as described^62^. After digestion with Turbo DNase (Ambion), 2 mg of RNA was retrotranscribed with Superscript III Reverse Transcriptase (Invitrogen) in 10 μl (final volume). A 0.5 μl aliquot of the reaction was PCR-amplified with primers 3969 and 3970 mapping within gp71 and gp73, respectively. Mock reverse transcription reactions (i.e., without Reverse transcriptase) were also PCR-amplified as a control for DNA contamination.

### Expression and purification of recombinant gp72 and gp73

The constructs were expressed in the LOBSTR *E. coli* expression strain (Kerafast) supplemented with either 30 μg/mL kanamycin for gp72-pET30b(+) or 50 μg/mL ampicillin for gp73-pET22b(+). The cultures were grown in L.B. medium at 37 °C until OD_600_ = ~ 0.3 when the temperature was dropped to 28 °C until an OD_600_ = ~ 0.6 and were induced with 0.5 mM IPTG for 2–4 h. For gp73, the protein was purified as described previously for P22 gp7^21^. Briefly, cell pellets were lysed by sonication in Lysis buffer (20 mM Tris-HCl pH 8.0, 300 mM NaCl, 4 mM MgCl_2_, 1% glycerol, 2 mM EDTA, 0.1% Triton X-100, 1 mM PMSF, 20 μg/mL DNase). After centrifugation at 18,000 rpm for 30 min, 4 °C, the insoluble fraction containing the protein was solubilized with rotation in Extraction buffer (20 mM Tris-HCl pH 8.0, 200 mM NaCl, 0.25–0.35% N-lauroylsarcosine, 1% glycerol, 20 μg/mL DNase) at room temperature for 1.5–2 h. The resulting supernatant, after centrifugation at 18,000 rpm for 30 min, 4 °C, was incubated with Nickel Agarose beads (GoldBio) for 2 h with rotation at 4°C. The beads were washed with Wash buffer (20 mM Tris-HCl pH 8.0, 200 mM NaCl, 0.025% DDM, 2 mM MgCl_2_, 1% glycerol, 1 mM PMSF, 5 mM imidazole) and eluted with Wash buffer containing 20–320 mM imidazole. The protein fractions were dialyzed against Dialysis buffer (20 mM Tris-HCl pH 8.0, 100 mM NaCl, 0.025% DDM, 2 mM MgCl_2_, 1% glycerol, 1 mM PMSF). The gp73 was further polished by SEC using an in-house packed Superose 12 16/60 column equilibrated with degassed Dialysis buffer. For gp72, the cell pellet was lysed by sonication in Lysis buffer (20 mM Tris-HCl pH 8.0, 25 mM NaCl, 4 mM MgCl_2_, 1% glycerol, 2 mM EDTA, 0.1% Triton X-100, 1 mM PMSF, 20 μg/mL DNase). The soluble portion, after centrifugation at 18,000 rpm for 30 min, 4°C was passed through Heparin (GE) column. The unbound fraction was collected and subjected to MonoQ (GE) and eluted with a buffer containing 1 M NaCl. Enriched fractions containing Gp72 eluted at ~ 550 nM NaCl was further purified by SEC using Superdex 200 16/600 (Cytiva). The protein used for Cryo-EM was further polished over Superose 6 10/300 GL (Cytiva). To form the gp72:gp73 complex, 90 μM gp72 was incubated with 180 μM gp73 in a total volume of ~ 1.2 mL for 3 h at room temperature. The volume was concentrated to 0.5 mL using 100 kDa MWCO (Vivaspin^®^ 6, Sartorius) concentrator and subjected to Superose 6 10/300 GL.

### Vitrification and data collection

2.5 μl of DEV mature virions at 1 × 10^12^ phages/ml were applied to a 200-mesh copper Quantifoil R 2/1 holey carbon grid (EMS) previously glow-discharged negatively for 60 sec at 15 mA using an easiGlow (PELCO). The grid was blotted for 7.5 sec at blot force 2 and vitrified immediately in liquid ethane using a Vitrobot Mark IV (FEI). Micrographs were screened on a 200 kV Glacios equipped with a Falcon4 detector at Thomas Jefferson University. EPU software was used for data collection using accurate positioning mode. For high-resolution data collection, micrographs were collected on a Titan Krios microscope operated at 300 kV and equipped with a K3 direct electron detector camera (Gatan) at the National Cryo-EM Facility at the Frederick National Laboratory, MD. Micrographs were collected in super-resolution mode plus energy filter at 20 eV with an image pixel size of 0.56 Å at 81,000x magnification, a nominal total dose of 50 e/Å^2^, 40 frames, and a defocus range − 0.8 to −1.6 μm. Further collection parameters are in Table 1.

### Cryo-EM single particle analysis

All steps of SPA were carried out using RELION 3.1.2^63,64^ and cryoSPARC ^65^software on a dual GPU Linux workstation. To reconstruct phage DEV, two sets of micrographs were combined after motion correction with MotionCor2^66^, yielding 17,245 micrographs. RELION’s implementation of motion correction was applied to the micrographs with options of ‘dose-weighted averaged micrographs’ and ‘sum of non-dose weighted power spectra’ every 4 e^−^/Å^2^. CTF (Contrast Transfer Function) estimation was carried out using CTFFIND4^67^. After 2D classification, symmetry-free low-res reconstruction, 3D classification, and 3D refinement in I3 symmetry, reference-free picked 109,000 particles were processed into two groups, HF capsids, and FF virions, with 8,000 and 16,000 particles, respectively. The particles of both groups were then expanded according to I3 symmetry using RELION’s *relion_particle_symmetry_expand* function to obtain expanded particles of 60 different orientations. A cylindrical mask (r = 200 Å, l = 300 Å) was created using SCIPION 3.0^68^ and then resampled onto the reference map from 3D refinement in I3 symmetry, covering the five-fold vertex on z-axis in Chimera^69^. The cylindrical mask was then used for symmetry-free sampling-free 3D classification to search for the tail. Locally aligned sub-particles were selected, extracted with z-axis shifted, and duplicates removed. The initial localized reference map was reconstructed directly from the selected particles using RELION’s *relion_reconstruct* routine. Selected particles were auto-refined using C5 symmetry, followed by 5-fold symmetry particle expansion. The 5-fold expanded particles were subjected to another round of sampling-free symmetry-free 3D classification aims for aligning ejecting components, portal, HT-adaptor, and tail tube at the unique 5-fold vertex. The capsid-portal aligned particles were then 3D refined with limited initial angular, CTF refined, and polished to generate localized maps with and without C12 symmetry applied. The same concept of localized reconstruction was applied to searching for scaffolding protein and the whisker ring (appendage), as well as improving coat protein resolution from 3.7 Å (I1 symmetry) to 3.3 Å (local C5 averaged). The final densities were sharpened using *phenix.auto_sharpen*^70^. Electron density maps were displayed using ChimeraX^71^. To reconstruct the structure of recombinant gp72 and the gp72:gp73 complex, all steps of SPA were carried out using cryoSPARC^65^ imposing C9 symmetry.

### De novo model building AlphaFold modeling and refinement

All *de novo* atomic models presented in this paper were built using Coot^72^ or Chimera^69^. We used five different maps for model building: (**i**) a 3.3 Å C5-averaged localized reconstruction of the mature head (**Supplementary Fig. 2A**) that revealed the atomic structure of the DEV coat protein (res. 1–399). (**ii**) a 3.1 Å C12-averaged localized reconstruction of the FF virion (**Supplementary Fig. 2A**) was used to build a model of dodecameric portal protein (res. 21–721) bound to twelve copies of the head-to-tail adaptor (res. 2–244), which also bound to twelve copies of the tail tube protein (2–158). The portal protein has no visible density of residues 1–21 in the C12 averaged map, but we were able to trace from residue #8 in the C5–C12 aligned asymmetric map; also, residues 722–726 of the C-terminal end of portal protein were not built due to missing densities. The HT-adaptor was fully modeled except for the first methionine. The C-terminal of the tail tube was not visible at the 3.1 Å resolution map after residue #158. The tail tube residues 159–321 were modeled in a low-resolution localized reconstruction of the full-length tail. (**iii**) a 4.0 Å C12-averaged localized reconstruction of the portal protein from the FF virion (**Supplementary Fig. 2A**) was used to identify and model gp72 residues 95–503 in the pre-ejection conformation. (**iv**) a 3.65 Å C9-averaged reconstruction (**Supplementary Fig. 2B**) was used to model recombinant gp72 (res. 46–224) in the post-ejection conformation. (**v**) A 3.15 Å C9-averaged reconstruction (**Supplementary Fig. 2C**) was used to build recombinant gp72 (res. 25–331) bound to gp73 (res. 1–155) in the post-ejection conformation. All atomic models were refined using several rounds of rigid-body, real-space, and B-factor refinement using *phenix.real_space_refinement*^73^ and validated using MolProbity^74^. Refinement statistics are in Table 1. The long- and short-tail fiber gp53 and gp56 prediction models were generated using AlphaFold^26^ and AlphaFold2^27^.

### Structure analysis and bioinformatics tools

All renderings of protein structures and map surface representations were generated using ChimeraX^71^. Model analysis and inspection were carried out in PyMol^75^. Structural comparison and identification were done using the DALI server^16^. Binding interfaces were analyzed using PISA^76^ and PDBsum^77^. RMSD between superimposed PDBs was calculated using SuperPose Version 1.0 (superpose.wishartlab.com)^78^. The Coulombic Electrostatic Potential was calculated and displayed with surface coloring using ChimeraX^71^. The identity of *Pseudomonas* phage vB_PaeP_DEV protein sequences (Taxonomy ID: 2034344, NCBI:txid2034344) was analyzed using bioinformatics tools, including blastp^79^ and searches in graphics mode of the NCBI Nucleotide section (GenBank MF490238.1). Sequence alignment was carried out in ebi.ac.uk/services^80^, including CLUSTAL O (1.2.4) for sequence alignment, EMBOSS Seqret for converting alignment format, and HMMER for homology search, and an in-house version of AlphaFold^26^ database for protein structure prediction. Sequence identity and similarity were carried out at Sequence Manipulation Suite (bioinformatics.org)^81^.

### Lipid Bilayer Measurements

Lipid bilayer experiments were performed using a silicon chip with a SU-8 aperture of 100 μm diameter^82^. The chip was placed in a *cis* part (grounded) of a custom-made cuvette. Each side of a SU-8 aperture was pre-treated with 0.5 μl of 5 mg/ml diphytanoylphosphatidylcholine (DPhPC; Avanti Polar Lipids) in hexane. After hexane evaporation, the cis part was mounted on a *trans* part of the cuvette, and both parts were filled with 600 μl of an electrolyte solution containing 1 M KCl, 10 mM HEPES (pH 7.4). Lipid bilayers were painted across the aperture with a solution of 20 mg/ml of DPhPC in n-decane. After membrane formation, a protein sample was added to the *cis* part of the cuvette. Baseline and detergent-containing buffers were examined to exclude contamination and detergent interference. Experiments were performed at + 100 mV potential. A pair of Ag/AgCl electrodes was inserted into the cuvette chambers and connected to an Axopatch 200 B patch-clamp amplifier (Molecular Devices). Currents were recorded at a sampling rate of 250 kHz, and low-pass filtered at 10 kHz. Data analysis was performed using Pythion, a custom software developed for analyzing current signals^82^. Pythion and SigmaPlot 11 (Systat Software) were used to generate the graphs shown in this study.

## Figures and Tables

**Table 1:** Map and model refinement statistics

Data Collection Statistics				
Specimen	*Pseudomonas* Phage DEV Virion			DEV gp72:gp73 Ejection Proteins
Facility/Microscope	NCEF/Titan Krios			SLAC-Stanford / Titan Krios
Detector	Gatan K3			Falcon 4
Imaging Software	SerialEM			EPU
Magnification	81,000 x			150,000 x
Voltage (kV)	300			300
Exposure (e^−^/Å^2^)	50			50
Exposure Time (sec)	3.2			12.9
Defocus range/step (μm)	−0.8 to −1.6 (0.2 increments)			−0.8 to −2.2 (0.2 increments)
Pixel size (Å)	1.12 (0.56)			0.56 (1.12)
Total movies (frames/movie)	17,245 (40)			4,897 (40)
Total dose (e-/Å^2^)	50			50
**Refinement Statistics**				
**Entry**	Major Capsid protein	Portal Protein/HT-adaptor/tail tube complex	gp72 pre-ejection conformation	gp72:gp73 post-ejection conformation
PDB /EMDB entry	8DTV/EMD-27709	7U4U/EMD-26335	8CZY/EMD-27102	8VXQ/EMD-43629
Initial particles number	109,000			310,000
Final particles number	19,000	16,000	16,000	61,000
Map Resolution (Å) at FSC _0.143_	3.3	3.1	4.0	3.15
Map Symmetry	C5	C12	C12	C9
Initial Model	*de novo*	*de novo*	*de novo*	*de novo*
Chains **/** Residues	9 / 3,591	36 / 13,212	12 / 4,740	18 / 4,140
Model-to-Map Correlation Coefficient (CC) *	0.89	0.89	0.70	0.78
MolProbity / Clash	1.7/5.1	2.0/10.9	2.0/13.2	1.6/3.2
R.M.S. deviations Bond Length (Å) / Angles (°)	0.002 (1) / 0.6 (15)	0.007 (0) / 0.7 (46)	0.002 (0) / 0.6 (0)	0.004 (3) / 0.7 (23)
Rotamer outliers (%)	0.1	0.9	0.0	0.4
Ramachandran (%) Fav / Allow / Outlier	91.5/8.4 / 0.1	92.2 / 7.6 / 0.2	94.6 / 5.4 / 0.0	93.2 / 6.7 / 0.1

* The model-to-map correlation coefficient CC is calculated in the map region around the model

**Table 2:** Bacteria, phages and plasmids

*Bacteria*		
Name	Relevant feature	Reference
PA01		83
PADR6	Frame shift mutation in *wzy*	11
PAER5b	Nonsense mutation in *wapH*	11
PAER6b	Frame shift mutation in *galU*	11
PAER10b	Nonsense mutation in *algC*	11
*Bacteriophages*		
Name	Relevant features^a^	Reference
DEV		8
DEV Δ53	Deletion 29656–32966; it eliminates gp53	This work
*Plasmids*		
Name	Relevant features^a^	Reference
pCas3cRh	It expresses the components of Type l-C CRISPR-Cas system	31
pCas3-01	pCas3cRh derivative carrying DEV 31196–31229 region; it expresses cr-RNA53 targeting gp53	This work
pCas3-09	pCas3-01 derivative carrying the two gp53 flanking regions with coordinates 29171 −29655 and 32967–33450	This work
pD53	pGM2148 derivative with 9 silent point mutations in the 31198-31230 region targeted by cr-RNA53	This work
pGM931	Shuttle vector carrying *araC-araBp* region	61
pGM2148	pGM931 derivative carrying the gp53-encoding 29656–32949 region	This work
gp72_pET30b(+)	untagged gp72 cloned in pET30b(+) between Ndel and Xhol	This work
gp73_pET22b(+)	gp73 cloned in pET22b(+) between Ndel and Xhol comprising C-terminal 6x his tag	This work

a Coordinates refer to Genbank MF490238.1

## Data Availability

Atomic coordinates for phage DEV coat protein (C5), portal:HT-adaptor:tail tube complex (C12), pre-ejection gp72 (C12), and gp72:gp73 complex (C9) have been deposited in the Protein Data Bank with accession codes 8DTV, 7U4U, 8CZY, and 8VXQ. The cryo-EM density maps have been deposited in the Electron Microscopy Data Bank with accession codes EMD-27709, EMD-26335, EMD-27102, and EMD-43629.
